# Assessing Viability and Stress Tolerance of Probiotics—A Review

**DOI:** 10.3389/fmicb.2021.818468

**Published:** 2022-01-27

**Authors:** Ulrika Wendel

**Affiliations:** Department of Analytical Development, Center of Excellence, BioGaia AB, Eslöv, Sweden

**Keywords:** probiotic, lactic acid bacteria, enumeration, viability, viable but not culturable, activity, microbiological methods, stress tolerance

## Abstract

The interest in probiotics has increased rapidly the latest years together with the global market for probiotic products. Consequently, establishing reliable microbiological methods for assuring the presence of a certain number of viable microorganisms in probiotic products has become increasingly important. To assure adequate numbers of viable cells, authorities are enquiring for information on viability rates within a certain shelf-life in colony forming units (CFU). This information is obtained from plate count enumeration, a method that enables detection of bacterial cells based on their ability to replicate. Although performing plate count enumeration is one manner of assessing viability, cells can still be viable without possessing the ability to replicate. Thus, to properly assess probiotic viability, further analysis of a broader group of characteristics using several types of methods is proposed. In addition to viability, it is crucial to identify how well the cells in a probiotic product can survive in the gastrointestinal tract (GIT) and thus be able to mediate the desired health benefit while passing through the human body. A broad spectrum of different assay designs for assessing probiotic gastric tolerance have been used in research and quality control. However, the absence of any consensus on how to assess these qualities makes it difficult to compare between laboratories and to translate the results into *in vivo* tolerance. This review presents and discusses the complexity of assuring that a probiotic is suitable for beneficial consumption. It summarizes the information that can be subtracted from the currently available methods for assessment of viability and stress tolerance of a probiotic, hereby altogether defined as “activity.” Strengths and limitations of the different methods are presented together with favorable method combinations. Finally, the importance of choosing a set of analyses that reveals the necessary aspects of probiotic activity for a certain product or application is emphasized.

## Introduction

The awareness of the health benefits of probiotic microorganisms, most often lactic acid bacteria (LAB), has increased rapidly the latest years and so has the global market for probiotic products (Rosenstiel and Stange, 2010; Hill et al., 2014; Hansen et al., 2018; Jackson et al., 2019; Fallico et al., 2020; Kiefer et al., 2020). Consequently, the market supply of probiotic products has become increasingly diverse, with a large number of both fermented food products and supplements, formulations, species, and strains (Kolaček et al., 2017).

The broad variety of products on the market makes it crucial that reliable methods are available for assuring probiotic viability (Kolaček et al., 2017; Hansen et al., 2018). Probiotics are a unique class of products since they consist of live organisms (Jackson et al., 2019). The term probiotic is defined by FAO/WHO as “live microorganisms that, when administered in adequate amounts, confer a health benefit on the host” (Rosenstiel and Stange, 2010; Hill et al., 2014). To assure that a probiotic product contains an adequate number of live organisms, authorities are asking for information on viability rates within a certain shelf-life in colony forming units (CFU) obtained from plate count enumeration (Hill et al., 2014; Fenster et al., 2019; Fiore et al., 2020; Hansen et al., 2020). The health effects of probiotics are reliant on dose and the minimum recommended amount to be consumed is often defined as 10^9^ CFU per day, labeled as CFU/ml or CFU/gram (Minelli and Benini, 2009). Measuring CFU by plate count enumeration is one manner of detecting live cells, but viability is more complex than solely defined by the ability to form colonies.

To properly assess viability, further analysis of a broader group of characteristics is required to obtain a better overview of the state of the probiotic. The fact that the products often consist of several strains, in some cases combined with additional active ingredients, and that the probiotic effect is specific for this specific combination, further complicates the viability analysis (Hill et al., 2014; Hansen et al., 2018).

As previously mentioned, the definition of probiotics is based on live microorganisms (Rosenstiel and Stange, 2010; Hill et al., 2014). This definition assumes that a large enough number of microorganisms also remain viable and survive the transit through the gastrointestinal tract (GIT), while facing stressors such as bile and gastric acid (Fredua-Agyeman and Gaisford, 2015). The numbers of viable microorganisms required to obtain a clinical effect is generally considered to be 10^6^ CFU/ml in the small bowel and 10^8^ CFU/g in the colon (Minelli and Benini, 2009). However, it is currently not required by authorities to account for analysis of tolerance against these stressors (Fiore et al., 2020). In addition, if tolerance assays are performed, it is often during strain selection and evaluation in the discovery and research phase, instead of after freeze-drying and formulation of the final probiotic product that will be offered to the consumer. The survival after exposure to bile and gastric acid can differ significantly, sometimes by several log units depending on formulation, freeze-drying and storage conditions (Saarela et al., 2009; Charnchai et al., 2016; Kim et al., 2018).

This review summarizes the information that can be subtracted from the currently available methods for determination of viability and stress tolerance of probiotics, here altogether defined as “activity”. It presents strengths and limitations of the different methods and discusses the importance of choosing a study design that reveals the whole picture of the activity of a probiotic.

## Viable but Not Culturable and the Great Plate Anomaly

The definition of bacterial viability has been debated in microbiology for a large period of time. Already in 1982, Xu et al. (1982) discovered that the waterborne pathogens that were subjects to their research were detected by direct viable counting but were not able to grow on plates. This additional population of cells had passed over to a viable but not culturable (VBNC) state, which lead to the questioning of the viability definition as the ability to form colonies. Three years later, Staley and Konopka (1985) coined the term “the great plate anomaly,” that has further on been used for describing the problem that a large portion of microorganisms in fact cannot be cultured under presently known conditions. The same authors concluded that these non-culturable bacteria indeed can be metabolically active.

So, what defines microbial viability? The question can without difficulty result in almost philosophical discussions. Although most researchers and scientists for long have agreed on that the definition is not limited to *in vitro* culturability, the answers to what defines microbial viability are not unambiguous and remains controversial. Through the years, several propositions on how to divide the non-culturable microbial population have been published (Kell et al., 1998; Breeuwer and Abee, 2000; Davey, 2011). One of the most recent definitions was published by Davis in 2014, who divided non-culturable microorganisms into the following states: (1) the non-replicating state (active physiology and membrane integrity), (2) the starving state (dramatic decrease in metabolism), (3) the dormant state (low metabolic activity and inability to divide without additional recovery attempts, VBNC), and (4) the irreparably damaged state (progressively declining metabolism that terminates in death).

There are a number of stresses in the manufacturing process and during storage, such as temperature shift-down, a lack of nutrients, and exposure to toxic agents such as hydrogen peroxide from autoclaved media, that can trigger the transfer of culturable populations into non-culturable states (Bogosian et al., 2000; Keer and Birch, 2003; Rittershaus et al., 2013). Lahtinen et al. (2006) showed in 2006 by flow cytometric (FC) analysis and plate count enumeration that three different probiotic strains lost their culturability during storage but maintained esterase activity, membrane integrity, and pH gradient across the cell membrane. Two years later, the same author showed that several *Bifidobacterium* strains, included in fermented food products, lost their culturability during storage but maintained high levels of rRNA and reductase activity (Lahtinen et al., 2008). Kramer et al. (2009) obtained similar results in 2009 when analyzing a lyophilized product containing both *Lactobacillus acidophilus* and *Bifidobacterium animalis* with polymerase chain reaction (PCR) and FC. The strains lost their culturability during storage but maintained their membrane integrity.

Besides the debate on the definition of viability, there has been several publications suggesting that non-viable probiotic microorganisms, sometimes referred to as parabiotics, also can provide benefits to the consumer (Taverniti and Guglielmetti, 2011; Fujiki et al., 2012; Lahtinen, 2012; de Almada et al., 2016; Piqué et al., 2019). However, parabiotics are outside the scope of this review.

## Stressors During the Lifetime of a Probiotic Product

**FIGURE 1:**
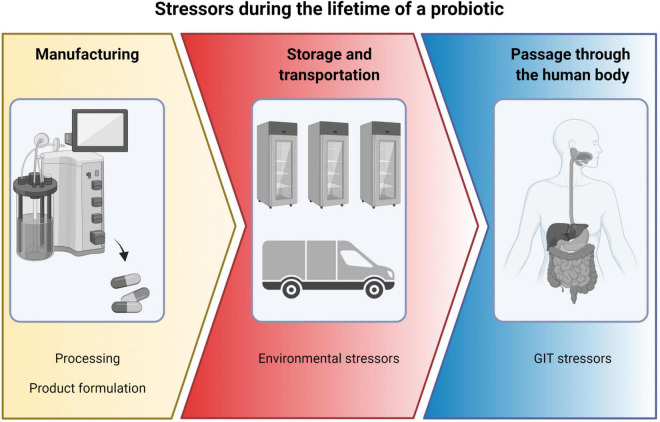
Overview of stressors during the lifetime of a probiotic, from manufacturing to storage and transportation and finally to passage through the human body. Created with BioRender.com.

A probiotic product must be able to tolerate exposure to a number of different stressors during its lifetime (Figure 1). Firstly, during manufacturing and secondly, during storage and transportation and finally, during its passage through the GIT.

### Manufacturing

After discovery of a beneficial probiotic strain, manufacturing is required before a probiotic product can be brought onto the market. The different operations included in manufacturing can affect many aspects such as survival, viability, and growth (Kolaček et al., 2017).

Firstly, the new strain must be able to retain its quality through scaled-up fermentation. In the scaled-up fermentation several factors can differ substantially from lab-scale, such as medium composition, cultivation time, pH, and gas atmosphere, which all can affect how well the cells tolerate later processing steps (Saarela et al., 2009; Kolaček et al., 2017; Stage et al., 2020). Other challenges in large-scale fermentations compared to initial lab-scale fermentations are keeping the same tight control, holding times and homogeneity (Fenster et al., 2019).

Secondly, most often a drying step is preferred for convenient further handling, such as freeze-drying (lyophilization) or spray-drying (Dolly et al., 2011). Drying is a manner of putting the cells into a resting, non-metabolic state for long-term preservation until consumption (Santivarangkna et al., 2008; Pegg et al., 2015; Broeckx et al., 2016; Hansen et al., 2018). Dehydration in general implicates shear stress and severe mechanical stress to the cell membrane which might lead to cell death (Santivarangkna et al., 2008; Iaconelli et al., 2015; Huang et al., 2018). The removal of water rapidly increases the ratio between cell surface area and cell volume, leading to membrane deformation. Increased contact of the cell membrane with the surrounding air imposes an increased risk for damage by reactive oxygen species.

Depending on the chosen method for drying, additional stresses are introduced. In freeze-drying, the initial formation of extracellular ice crystals leads to an increasing concentration of medium solutes, resulting in osmotic stress (Broeckx et al., 2016; Obruca et al., 2016). Ice crystals are also formed intracellularly, possibly leading to membrane destruction and organelle disruption (Fuller, 2004). In addition, ice crystal formation can lead to increased concentrations of reactive oxygen species (Baek and Skinner, 2012). In spray-drying, heat is the main stressor, affecting a large part of the cellular components (Santivarangkna et al., 2008; Broeckx et al., 2016). Although the exact inactivation mechanisms in spray drying are not fully understood, exposure to thermal stress can lead to denaturation of proteins and destabilization of membranes. Ribosomal damage might be the critical component in heat inactivation.

Although drying provides efficient protection of the cells, the following product formulation exposes the bacteria to different stresses, weather it is inclusion into a food product or into a supplement.

### Transportation and Storage

After manufacturing, the probiotic products must be transported from the manufacturing site and survive storage in given conditions in periods of time often exceeding 12 months (Fiore et al., 2020). During storage, the bacterial cells can be exposed to environmental stressors such as temperature, water, oxidation, pH, and light (Santivarangkna et al., 2008; Pegg et al., 2015; Broeckx et al., 2016; Kolaček et al., 2017; Fenster et al., 2019; Fiore et al., 2020). One of the undesired effects during storage is membrane lipid oxidation (Santivarangkna et al., 2008). For dried cells, the relative humidity is of high importance to be able to remain in the protecting dried state. In general, low temperatures and low humidity contributes to higher survival rates during storage.

### Passage Through the Human Body

In addition to coping with the different stresses of manufacturing, storage, and transportation, the final probiotic product should be able to survive and maintain its activity while passing through the human body. Either before or during consumption, depending on the product type, the dried cells must undergo rehydration. Rehydration is challenging for the cell membrane, since it includes moving from a gel-like state into a liquid crystalline (Broeckx et al., 2016).

After rehydration, the bacterial cells must survive through the challenging upper GIT (Derrien and van Hylckama Vlieg, 2015). The upper GIT offers a number of trials to the ingested probiotic. The first challenge is passage through the stomach, where the gastric juice exposes bacteria to low pH (< 3) and high concentrations of pepsin (Derrien and van Hylckama Vlieg, 2015). Both these two stressors can lead to cell inactivation and death. However, the pH, composition, buffering capacity and volume of the gastric juice, and the transit time are largely affected by the recent food consumption history. In addition, survival through the GIT is highly strain dependent and depends on the product matrix, e.g., powder or capsule (Fredua-Agyeman and Gaisford, 2015).

The second challenge is the passage through the duodenal loop of the small intestine where the main bile salt exposure occurs (Taranto et al., 2003). Bile is more harmful to bacteria than low pH since it acts as a detergent and thus disrupts the membrane (Ilango et al., 2016). Exposure to bile changes the lipids in the cell membrane, possibly affecting both cell permeability and the interactions between the membrane and its environment (Taranto et al., 2003). As highlighted by Bustos et al. (2012) differences in bile tolerance between strains are also reliant on the ability to express bile salt hydrolase enzyme. LAB strains that are bile salt hydrolase positive are more tolerant to the consequences of bile exposure (Bustos et al., 2012). The ability to hydrolyze bile salts is considered as one of the key features of probiotic bacteria according to the World Health Organization, although far from all probiotics have this ability (WHO and FAO, 2006). In addition to bile salt hydrolysis, LAB strains can possess other abilities to cope with bile exposure such as active efflux of bile salts and changes in the cell wall and cell membrane composition (Ruiz et al., 2013). Apart from bile salts, the small intestine also contains pancreatin and lipase, that as well are potentially harmful to bacteria (Derrien and van Hylckama Vlieg, 2015).

In addition to surviving the passage through the human body, the probiotic bacteria must be in a sufficient state for being able to affiliate beneficial health effects. Syntrophic interactions with the existing microbiota, enhancement of the epithelial barrier, immunomodulation, secretion of antimicrobial substances, prolonged persistence in the gut by colonization, and the ability to form biofilms, are factors that are associated with probiotic function (Bermudez-Brito et al., 2012; Frese et al., 2013; Segers and Lebeer, 2014; Li et al., 2021). Mucus-binding pili, that enables both host interactions, adherence, and biofilm formation, and that might be involved in immunomodulatory interactions, can be expressed to a lesser extent by a challenging host environment or be damaged by exposure to for example shear stress (Segers and Lebeer, 2014).

## Manners of Assessing the Activity of Probiotics

**FIGURE 2:**
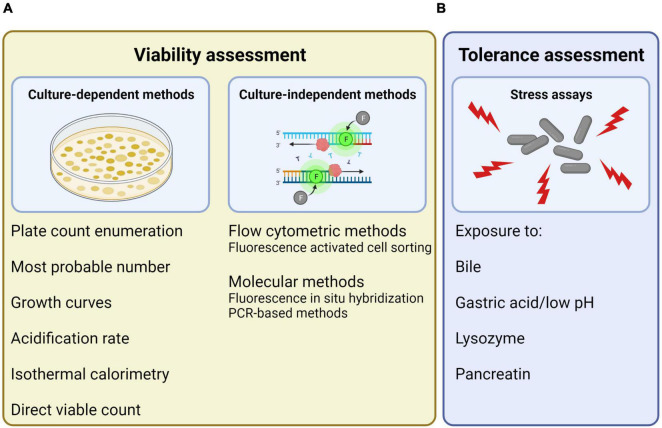
Overview of different manners of assessing probiotic activity, here divided into **(A)** viability assessment, which can be performed using both culture-dependent and culture-independent methods, and **(B)** tolerance assessment, which can be performed by exposure to different stressors. Created with BioRender.com.

The manners of assessing the activity of probiotics have here been divided into viability assessment (Figure 2A) and stress tolerance assessment (Figure 2B). The described methods for viability assessment have been further divided into two subcategories: (1) culture-dependent methods, that are based on the ability of the cells to replicate, and (2) culture-independent methods, that are based on other cell characteristics than the ability to replicate.

### Culture-Dependent Methods for Viability Assessment

Culture-dependent methods, where plate count enumeration is the most prevalent, have for long been the standard for viability measurement of probiotic cultures and products (Lahtinen et al., 2006; Davis, 2014; Hansen et al., 2020; Kiefer et al., 2020). They are uncomplicated to perform but laborious and require media and conditions that are well adapted to the strain of interest (Davis, 2014). Culture media are always, to a certain extent, selective for a certain type of bacteria and might be unequally optimal between bacteria from different genera or even between different strains (Coeuret et al., 2003). Factors such as oxygen tolerance, antibiotic susceptibility, and nutritional preferences must be considered when choosing and designing suitable media and cultivation conditions. Thus, although sometimes possible with antibiotics, it can be difficult to separate a mixture of two or several strains with highly similar growth requirements (Jackson et al., 2002; Hansen et al., 2018; Vinderola et al., 2019; Kiefer et al., 2020).

#### Plate Count Enumeration

There are multiple ways of performing plate count enumeration. In brief, the probiotic sample is dissolved/suspended in a buffer/medium and diluted to an appropriate concentration. The resulting solution is then distributed either onto a petri dish filled with solid agar medium or mixed with melted agar medium and let to solidify in a petri dish. After incubation under suitable conditions, the colonies that have been formed can be counted and approximated to represent the number of viable cells present in the original sample.

There are several advantages of using plate count techniques for assessing the viability of a probiotics. For example, low costs are required, they enable detection of proven viability and ability to replicate, are easy to set up in a laboratory, and give highly visual results, allowing observation of colony morphologies and potential contamination. In addition, since these methods have traditionally been the established way of performing microbiological viability assessments, the available published historic data on their various applications are extensive. Specifications of the viability of probiotic products are also inquired for in CFU from authorities (Hill et al., 2014; Fenster et al., 2019; Fiore et al., 2020; Hansen et al., 2020). The International Organization for Standardization (ISO) offers standardized plate count enumeration protocols for probiotics, both for mesophilic lactic acid bacteria in general, ISO 15214 (ISO, 1998), for *Lactobacillus* acidophilus, ISO 20128 (ISO, 2006) and for presumptive bifidobacteria, ISO 29981 (ISO, 2010).

Although widely used, plate count enumeration methods for viability assessments comes with several limitations. Firstly, culturing bacteria on agar plates is highly time-consuming, mostly due to preparation of media and long incubation times (Fredua-Agyeman and Gaisford, 2015; Vinderola et al., 2019). Secondly, plate count enumeration is based on the ability of the cells to form colonies by replication. However, as previously mentioned, the definition of viability includes more abilities than replication (Xu et al., 1982). Plate count enumeration is also based on the assumption that only one cell will form one colony. However, during manufacturing of a culture powder, there is a risk for clumping (Davis, 2014; Vinderola et al., 2019). The clumping can give rise to uneven spreading of the cells during plating, which might result in one colony being formed by multiple cells. Plate count enumeration of bacterial cells exposed to stress conditions e.g., due to freeze-drying and manufacturing, can also affect culturability (Olszewska et al., 2019). In general, plate count methods are associated with underestimations and large variations, sometimes reaching up to 35% (Kogure et al., 1979; Olszewska et al., 2019). This can partly be explained by media quality, fluctuations in growth conditions, and inherent errors in serial dilutions in the sample preparation (Kogure et al., 1979; Corry et al., 2007; Hansen et al., 2020).

#### Most Probable Number

The most probable number method is based on that a liquid medium turns turbid during bacterial growth (Bibiloni et al., 2001; Sutton, 2010; Angelov, 2014). By making a 10-fold serial dilution of a bacterial cell solution in replicates, the point will be reached where the solution is so diluted that only a few of the replicates of a certain dilution give rise to turbidity. The number of tubes with confirmed turbidity, thus growth, at the different dilutions can be used to estimate the number of viable cells in the original sample by utilizing premade tables based on statistics using 95% confidence intervals. Although not traditionally used for viability measurements in probiotics, there are a few examples (Bibiloni et al., 2001; Angelov, 2014).

The most probable number methodology is based on several assumptions (Blodgett, 2020). These assumptions are listed in the FDA Bacteriological Analytical Manual (BAM), namely: random bacterial distribution within the sample, that the bacteria exist as separate units without clustering and repelling, that growth from as few as one single cell will be detected, and that individual tubes are treated as independent samples.

Although often used when handling very diluted samples, which is often not the case with probiotics, this method presents an alternative to plate count enumeration when facing problems with colony formation e.g., from a disturbing matrix (Sutton, 2010; Blodgett, 2020). However, it is important to remember that results obtained from this method are only the statistical probability of the number of replicating bacteria in a sample, compared to for example plate count enumeration that results in an actual number.

#### Growth Curves

Growth curves are created by culturing bacterial cells in a liquid medium and continuously measuring the increase in optical density (OD) as the cells replicate and then plotting the measured OD values against time. The resulting curves can reveal a number of different characteristics. Factors especially interesting for assessing replication potential are the maximum growth rate, μ_max_, and the duration of the lag phase, t_lag_ (Bircher et al., 2018). It has been observed that t_lag_ negatively correlates with viable cell counts, in that particular case obtained with the most probable number method, and that previous manufacturing and storage conditions, such as freezing, freeze-drying, and spray-drying, have an impact on both generation times and growth initiation (Squires and Hartsell, 1955; Iaconelli et al., 2015; Lipson, 2015; Bircher et al., 2018). Indications of low viability, as interpreted from growth curves, may have a direct link to metabolic activity in the GIT (Bircher et al., 2018). However, further research is called for to fully elucidate the relationship between μ_max_, t_lag_ and viability.

One issue in growth curve viability assessment is how to decide the starting viable cell count when comparing different cell samples. Whether you choose to start your growth curve with a sample concentration based on OD, membrane integrity measured by FC, or on CFU from plate count enumeration, can seriously affect your result. Which method that is the most suitable for deciding the start concentration of a cell sample for a growth curve, can be debated. Starting at the same OD might introduce uncertainty from differences in the amount of non-viable cells and debris between samples (Beal et al., 2020). CFU from plate count enumeration is based on replication, as is growth curve assessment. However, plate count enumeration results in a cell number based on the ability to replicate on an agar plate at given conditions, which is not the same conditions as in the growth curve assessment. Starting at a concentration based on live/dead assessment with FC (described later) can reveal a live population that might be able to replicate during growth curve assessment. However, FC results also reveal a damaged cell population, of which the part that is able to replicate in the given conditions is unknown.

#### Acidification Rate

Acidification rate analysis, performed by simply measuring the change in pH in a liquid medium culture with time, is most commonly used for assessing the quality of milk starter cultures containing LAB (Fonseca et al., 2000). For this application, an ISO method is available, ISO 26323 (ISO, 2009). However, the amount of produced lactic acid might also be used as a complement or substitute to other viability measurements in a variety of samples containing probiotic LAB (Saez-Lara et al., 2015). Fonseca et al. (2000) used acidification rate to compare the resistance to freezing between a number of different LAB strains and could see that the acidification rate was proportional to cell concentration under well-defined experimental conditions. Poor reliability in plate count enumeration due to chain building can be circumvented by instead performing acidification rate measurements.

#### Isothermal Microcalorimetry

Isothermal microcalorimetry is a technology that is based on that heat is produced by a replicating, metabolizing bacterial population (Mihhalevski et al., 2011; Garcia et al., 2017; Fredua-Agyeman and Gaisford, 2019; Nykyri et al., 2019). The heat production is approximately 2 pW per cell, enabling measurements in bacterial samples with cell concentrations from approximately 106 CFU/ml (Fredua-Agyeman and Gaisford, 2019). The heat production is proportional to the number of bacterial cells in a sample and can be plotted as μW against time. For the most simple isothermal microcalorimetry measurements, a bacterial sample is placed in a glass ampoule. The heat production is then recorded for as long as it occurs (Braissant et al., 2010). By following the heat production of the bacterial population, it is possible to obtain real-time, continuous viability data, in contrast to for example plate count enumeration that only gives the endpoint of the viability measurement (Braissant et al., 2010; Fredua-Agyeman and Gaisford, 2019). From the heat measurement, several different growth specific data can be estimated, such as biomass yield and specific growth rate (Mihhalevski et al., 2011). Since the heat recordings are passive, the bacterial sample is available for further analysis (Braissant et al., 2010).

Apart from the above-mentioned advantages of isothermal microcalorimetry, the technology is appreciated for not requiring laborious sample preparation steps, for giving results within a few hours, and for requiring very low running costs (von Ah et al., 2008; Entenza et al., 2014; Garcia et al., 2017). The method enables high sensitivity measurements due to the low detection levels of the produced heat and the limited scattering of the data, thus, providing viability data with high accuracy and low standard errors (Braissant et al., 2010; Garcia et al., 2017). Exposure to stressors such as drying followed by rehydration does not seem to affect isothermal microcalorimetry results.

Despite the many advantages of using isothermal microcalorimetry for viability measurement of probiotic cultures, this method also has a number of drawbacks. For example, it requires careful calibration with a reference sample at the exact temperature of measurement in order to obtain high accuracy and sensitivity (Braissant et al., 2010). In addition, since measurement of heat is non-specific for bacterial growth, it cannot be excluded that other cellular processes are contributing to the resulting signal. Finally, isothermal microcalorimetry is still a technology that is not as established for enumeration of probiotic cultures, as for example Plate count enumeration or PCR techniques. Consequently, the availability of large studies and comparative material is limited.

#### Direct Viable Count

Direct viable count is a direct counting technique that enables differentiation between viable and non-viable cells by their ability to replicate. By incubating the bacterial sample with nutrients and a gyrase inhibitor, cell division is inhibited, resulting in viable cells to elongate and become considerably larger than non-viable cells (Kogure et al., 1979; García-Hernández et al., 2012). Viable cells can then be distinguished from non-viable cells merely on size during microscopic observation.

Direct viable count is a method that requires a relatively small amount of time for sample preparations and that enables simple counting using a microscope. On the other hand, examination by the eye is often necessary, which puts requirements on the skills of the technician performing the analysis and compromises efficacy.

### Culture-Independent Methods for Viability Assessment

The methods included in this section are divided into flow cytometric methods and molecular methods. The described methods all have in common that they do not require that the microorganisms have the ability to replicate in order to be detected.

#### Flow Cytometric Methods

Although first developed for the hematology field, FC has become an increasingly used tool within microbiology (Davis, 2014). FC is a technology that enables cell-by-cell observation of up to millions of cells and division of these into subpopulations based on different characteristics (Maciorowski et al., 2017). Briefly, the cell sample is passed through a so-called sheath fluid stream, centering single cells (Shapiro, 2003; Tracy et al., 2010; Adan et al., 2016). The centered cells are hit by a light source on their passage, most often a laser beam, that will result in light scattering or in excitation of any applied fluorescent probes. Optical detection of light scattering reveals morphological and structural cell characteristics while detection of the emission from excited fluorescent probes can give information of specific cell properties or cell components. The optical signal is transmitted to an electronic network that converts it to digital data that enables computational recordings of the light intensity.

**FIGURE 3:**
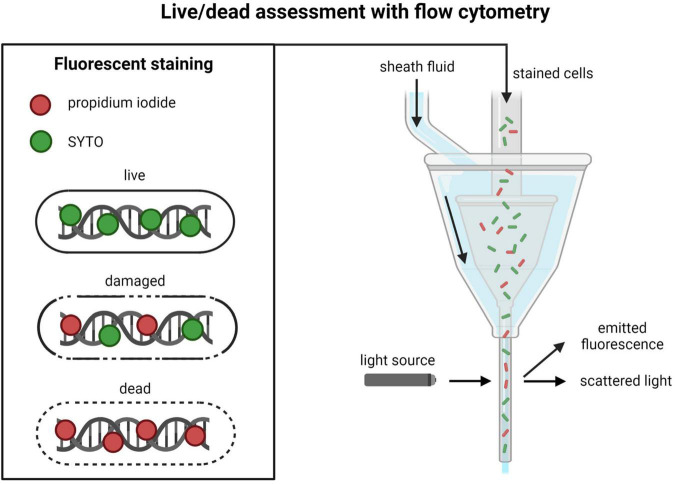
Overview of live/dead assessment with propidium iodide/SYTO staining and flow cytometry. Created with BioRender.com.

In microbial viability FC measurements (often called live/dead assessment), dead cells are most commonly distinguished from live cells by determining membrane integrity using the DNA stains propidium iodide (PI) and SYTO (Figure 3). SYTO will enter all cells, while PI only will enter cells with compromised and permeable membranes (Díaz et al., 2010; Tracy et al., 2010). The resulting information from these two stains is used for dividing the total cell population into one live (impermeable) and one dead (permeable) subpopulation (Breeuwer and Abee, 2000). Apart from these two subpopulations, it is possible to detect cells in intermediate states that are not completely permeable to PI but will be stained to a smaller extent (Olszewska et al., 2019). This subpopulation is often interpreted as damaged cells. Apart from PI and SYTO staining, there are a wide variety of different fluorescent probes, or fluorochromes, that can be used for detecting different cell properties and components (Adan et al., 2016). The available ISO method for viability measurement of LAB in probiotics, ISO 19344, contains protocols for determination of enzymatic activity, membrane integrity, and metabolic activity (ISO, 2015).

There are many advantages of using FC and fluorescent probes for assessing probiotic activity. The use of fluorescent probes offers a high sensitivity and the ability to observe several parameters simultaneously (Shapiro, 2003; Lahtinen et al., 2006; Díaz et al., 2010; ISO, 2015). The instrument also allows high-throughput analysis in the regions of several thousands of cells per second (Davis, 2014). In addition, FC indicates the heterogeneity of a population, which can be valuable information for bacterial cultures intended for probiotic products (Tracy et al., 2010). Compared to for example plate count enumeration, the time required from sample preparation to result is considerably shorter (Breeuwer and Abee, 2000).

Despite providing very detailed information about all cells in sample, FC results are presented on a population basis and not as single cells (Olszewska et al., 2016). This requires certain knowledge of the cell population to be able to achieve an experimental design that will yield the maximal amount of information. If using several fluorescent probes at the same time, optimization of any potential overlaps between their emission spectra must be performed to avoid misinterpretations (Adan et al., 2016). In addition, if using fluorescent labeling of the cells, bacterial autofluorescence may disturb the results (Moter and Göbel, 2000). As in plate count enumeration, FC results can be affected by clumping, although it is often possible to identify an inhomogeneous population (Kramer et al., 2009). FC detects phenotypic differences, which can be a limitation when differentiating between strains with only small genetic differences. This presents a problem when analyzing probiotic products containing multiple strains.

##### Fluorescence Activated Cell Sorting

Fluorescence activated cell sorting (FACS) is a technology that enables separation of the sample analyzed by FC into the detected subpopulations and collection of these for further analysis (Tracy et al., 2010). This additional feature allows observation of for example culturability in connection to the different characteristics detected in the FC analysis (Olszewska et al., 2019).

Although taking the FC analysis one step further, one limitation of FACS is the risk of damaging the sorted cells during measurement by e.g., laser exposure, shear stress and mechanical sorting (Olszewska et al., 2019). Thus, there is a risk that for instance the culturability of a FACS subpopulation might be compromised.

#### Molecular Methods

Usage of molecular methods, which are based on nucleic acid detection, enables detection and in combination with identification of bacteria on a strain-level with high specificity and sensitivity in a high-throughput manner (Keer and Birch, 2003). Therefore, molecular methods are of great use in samples or products containing multiple probiotic strains (Keer and Birch, 2003; Postollec et al., 2011; Hansen et al., 2018; Kiefer et al., 2020).

However, nucleic acid levels alone are not considered to be a reliable viability marker (Keer and Birch, 2003; Amor et al., 2007; Kiefer et al., 2020). Nucleic acid content was previously considered to distinguish live cells from dead cells because of post-death degradation (Keer and Birch, 2003; Nogva et al., 2003). However, the DNA molecule is very stable and can persist in cells even after death has occurred. The RNA molecule on the other hand, is very unstable and is therefore not optimal to be the base of high accuracy live/dead measurements. In addition, in live cells, both growth rate and placement in the cell cycle can give large variations in nucleic acid levels (Moter and Göbel, 2000; Bouvier and Del Giorgio, 2003). However, the strength of molecular methods to identify specific probiotic species and strains, makes them optimal to use on combination with other methods detecting viability markers, or in combination with viability dyes for strain-selective detection of viable cells.

##### Fluorescent in situ Hybridization

Fluorescent *in situ* hybridization is a technique where hybridization of bacterial cells with complementary fluorescence-labeled oligonucleotide probes is used, enabling distinction between groups of bacteria in a mixed population (Amann et al., 1990, 1995; Coeuret et al., 2003). Depending on the choice of probe, the groups of bacteria can be separated on species or even on strain level (Coeuret et al., 2003). Fluorescent probes with different emission wavelengths can be used on the same sample, enabling simultaneous detection of several sequences (Moter and Göbel, 2000). In addition, fluorescent *in situ* hybridization does not require elaborate sample preparation steps and is relatively fast to perform (Moter and Göbel, 2000; Blasco et al., 2003). This method also offers a very high sensitivity if using a carefully designed probe. Fluorescent *in situ* hybridization does not itself provide information on viability but can be successfully combined with e.g., microscopy, FC, and direct viable count for further information on strain-specific viability (Amann et al., 1990; Moter and Göbel, 2000; Blasco et al., 2003; Ercolini et al., 2006; Tracy et al., 2010; García-Hernández et al., 2012; Olszewska et al., 2016).

Problems may arise due to insufficient optimization of permeabilization treatment prior to hybridization (Moter and Göbel, 2000). As any other fluorescence-based technology, autofluorescence from the analyzed bacteria can also compromise the reliability of the results and decrease the signal-to-noise ratio. Matrix-dependent differences have been observed when using this method, emphasizing the importance of careful evaluation of the influence of the matrix when setting up a fluorescent *in situ* hybridization method (Davis, 2014).

##### Polymerase Chain Reaction-Based Methods

There are several molecular methods used for detection of probiotic bacteria that are based on PCR. Alone, usage of these methods does not enable selective quantification of genetic material only from viable cells. However, it is possible in combination with the nucleic acid-intercalating dyes ethidium monoazide (EMA) and propidium monoazide (PMA).

###### Real-Time Quantitative Polymerase Chain Reaction

Real-time quantitative PCR (qPCR) enables identification and quantification of microorganisms on strain level (Kramer et al., 2009; Postollec et al., 2011; Villarreal et al., 2013). In brief, a specific DNA sequence is enzymatically amplified by a thermostable DNA polymerase that attaches to pre-designed oligonucleotide primers that have hybridized to the template DNA (Postollec et al., 2011). Variation of the reaction temperature enables denaturation of the two DNA strands, hybridization of the primer, attachment of the polymerase and replication of the DNA sequence, followed by polymerase detachment. Repetition of this chain of events in cycles results in an exponential amplification of the targeted DNA sequence. In qPCR, a fluorescent reporter probe is used for monitoring the increasing nucleic acid levels. There are two different reporter probes that are typically used—the DNA-binding SYBR ^®^ Green and TaqMan ^®^ (Applied Biosystems), that is used for the hydrolysis probe method (5′ nuclease assay) (Gibson et al., 1996; Wittwer et al., 1997; Postollec et al., 2011). SYBR ^®^ Green is not specific for a certain DNA sequence and can therefore offer the flexibility of being used in many different setups of genetic detection. TaqMan ^®^ on the other hand is used for detection of particular amplicons and therefore results in a higher specificity. By plotting the fluorescence of the probes against the number of cycles, quantification can be achieved.

If performed with thorough knowledge and optimized protocols, qPCR technology is rapid, offers high specificity and sensitivity, and can distinguish between probiotic strains with large genetic similarities (Bustin et al., 2005; Postollec et al., 2011). In addition, both the sample preparation steps, and the analysis are possible to automate (Postollec et al., 2011).

Although qPCR can identify a certain organism on the molecular level, quantification requires a previously optimized standard curve, based on purified DNA (Hansen et al., 2018). There are many commercially available kits for DNA purification, but usage of these does not always result in complete extraction. In addition, the PCR amplification reaction is sensitive to inhibitors (Gobert et al., 2018). Amplification must result in DNA concentrations above the background level of the standard curve, which limits the possibilities of quantification of genetic material present in very low concentrations (Hindson et al., 2011). For a reliable qPCR protocol, proper positive and negative controls must be analyzed to exclude contamination (Bustin et al., 2005).

###### Digital Polymerase Chain Reaction

Digital PCR (dPCR) is a PCR method where the sample is extensively diluted and then divided into separate minireactors, as droplets or in a chip-based manner (Hindson et al., 2011; Huggett et al., 2013; Hansen et al., 2018; Kiefer et al., 2020). Consequently, a number of the minireactors will not contain the target sequence due to the high dilution grade. The PCR amplification reactions take place within the minireactors and the number of positive (generated from TaqMan ^®^ chemistry) and negative signals from the separate reactions are calculated and translated to gene copies per μl using Poisson statistics.

Similar to qPCR, dPCR can quantitatively distinguish between highly similar probiotic strains, with high precision within a short amount of time (Hansen et al., 2018). However, in opposite to traditional qPCR technologies, dPCR enables absolute quantification of DNA sequence copy numbers, which are not based on a standard curve using purified DNA (Hindson et al., 2011; Hansen et al., 2018). Thus, the dPCR approach is less sensitive to inhibitors compared to qPCR and can detect lower concentrations of genetic material (Huggett et al., 2013; Gobert et al., 2018).

However, similar to qPCR, dPCR also requires careful design and optimized controls to be able to deliver robust results (Huggett et al., 2013). In addition, although dPCR presents many advantages compared to qPCR, it is still more expensive, and requires more advanced technology and complicated workflows (Hindson et al., 2011; Gobert et al., 2018). Although it could be argued that dPCR results are built on estimations, limiting their credibility compared to absolute values, the very large number of minireactors enables statistical calculations with very high precision (Hindson et al., 2011).

###### Polymerase Chain Reaction-Based Methods in Combination With Ethidium Monoazide and Propidium Monoazide Staining

**FIGURE 4:**
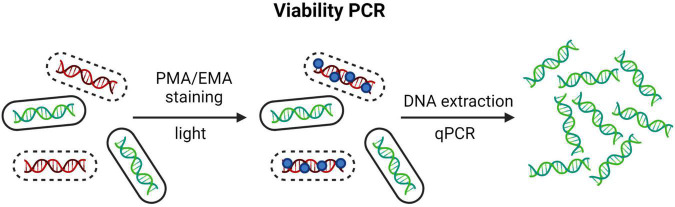
The principle of qPCR combined with PMA/EMA chemistry, so called viability PCR. Created with BioRender.com.

The inability of molecular methods to selectively quantify genetic material only from viable cells was circumvented by Nogva et al. (2003) in 2003 by utilizing staining with EMA and later by Nocker et al. (2007) in 2007 with PMA, both in combination with PCR technology. These dyes are nucleic acid-intercalating and binds irreversibly upon photoactivation, thus strongly inhibiting further amplification attempts by PCR (Nogva et al., 2003; Nocker et al., 2007; Fittipaldi et al., 2012). EMA is membrane-permeable, and PMA is membrane-impermeable. Hence, these stains enable selective amplification of genetic material from cells with intact membranes. PCR methods utilizing PMA and EMA staining, are often referred to as viability PCR, or v-PCR (Figure 4). Both qPCR and dPCR have been used for separation of viable and non-viable bacteria on a strain level with PMA/EMA chemistry (Rudi et al., 2005; Kramer et al., 2009; Gobert et al., 2018; Kiefer et al., 2020).

Despite revolutionizing PCR technology by extending molecular detection with membrane permeability markers, EMA and PMA chemistry does have its limitations. Several publications have addressed differences in permeability to these dyes depending on a collection of factors (Fittipaldi et al., 2012; Gobert et al., 2018; Kiefer et al., 2020). Influencing factors are dye concentration, physicochemical properties of the matrix, interplay between permeability and active efflux systems, differences in the compositions of cell membrane and cell walls, cytotoxicity of the dyes, cell concentration, and sample ratio between live and dead cells. In other words, careful optimization and use of controls are crucial when performing viability detection with PMA/EMA.

### Stress Tolerance Assessment

Apart from remaining viable in the probiotic culture or product, the included microorganisms must also have the ability to survive the passage through the GIT. To assess this ability, many different setups of both culture-dependent and culture-independent assays have been presented that are designed to reveal probiotic survival in the presence of different stress factors. The most prevalent stress factors used in tolerance assays are bile and gastric acid/low pH (with or without pepsin, a proteolytic enzyme secreted in stomach), representing the passage through the stomach and the duodenum, respectively (Saarela et al., 2005). Exposure to lysozyme (found in saliva) and pancreatin (present in the small intestine) are also included in a few published studies.

Although widely used, there is no consensus or standardized manner on how to perform stress assays to estimate the survival of probiotics during their passage through the human body. A variety of stressors and different compositions of these are used, and the resulting tolerance is analyzed with different methods. In some cases, the survival after an exposure to a stress factor is assessed and in other cases the growth in the presence of a stress factor is the target of observation. Tables 1–4 summarize the conditions in a selection of published stress assays assessing probiotic tolerance against gastric acid/pH/pepsin, bile, pancreatin, and lysozyme. The designs of these assays are highly varying, both in terms of tested material (fresh cells/freeze-dried culture, spray-dried culture), type of stressor (artificial, biological), physiological or non-physiological concentrations, exposure times, and chosen methods for assessing the effects of the stress exposure on the bacteria. The assay design of course has a large impact on the results, thus making different assay setups difficult to compare. Saarela et al. (2005) highlighted in 2005 the consequences of using different types of bile in a tolerance assay on *Bifidobacterium animalis* ssp. *lactis* cells. Bile extract was severely more harmful to the cells than bile acid. There is also a batch-to-batch variation to consider when using biological bile and differences depending on species of origin. Fredua-Agyeman and Gaisford also pointed out in a publication from 2015 that simulated gastric conditions in stress assays often present a very poor similarity to the complex environment that probiotic bacteria are exposed to in the GIT. For example, the bile salt concentration in the gut is varying over time and in the different parts of the intestine (Marteau et al., 1997). In addition, *in vivo*, the combined stresses of exposure to e.g., bile and gastric acid, can be much more damaging than in an *in vitro* assay only exposing the cells to one stressor. Continuous exposure to bile, as seen *in vivo*, is more detrimental compared to a shock exposure for a short period of time, as often seen *in vitro* (Taranto et al., 2003). All of these factors have to be considered when designing a stress assay.

**TABLE 1:** A summary of the details of a collection of published pH tolerance assays.

Genus	Material	pH	Duration (h)	Detection	References
*L. rhamnosus*	Freeze-dried	2.5	1	Plate count enumeration	Stage et al., 2020
*L. casei*	Fresh cells	2	1	Plate count enumeration	Huang et al., 2018
Different LAB	Fresh cells	2.5	3 and 6	Plate count enumeration	Ilango et al., 2016
Different LAB	Spray-dried	2	0.5, 1, 1.5, and 2	Plate count enumeration	Ilango et al., 2016
Different LAB	Freeze-dried	2	0.5, 1, 1.5, and 2	Plate count enumeration	Ilango et al., 2016
*B. animalis*	Fresh cells	2 and 3, with or without pepsin	3	Plate count enumeration	Charnchai et al., 2016
*L. plantarum*	Spray-dried	2, with pepsin	1, 2, 3, and 4	Plate count enumeration	Dolly et al., 2011
*L. plantarum*	Freeze-dried	2, with pepsin	1, 2, 3, and 4	Plate count enumeration	Dolly et al., 2011
Different LAB	Fresh cells	2.5	0.5, 1, 1.5, 2, 3, 4, 5, 6, 7, and 24	OD_620_	Jacobsen et al., 1999
Different LAB	Fresh cells	2.5	4	Plate count enumeration	Jacobsen et al., 1999
Different LAB	Fresh cells	3, 4, 5, 7, 9, 11, 13	1, 2, 3, 4, 5, 6, 7, 8, 9, 10, 11	OD_600_	Brink et al., 2006
*L. plantarum*	Fresh cells	2, 2.5, and 3	0.5	Plate count enumeration	Turchi et al., 2013
*L. casei*	Spray-dried	2.0, 3.0, and 4.0	0.5, 1, 2, and 3	Plate count enumeration	Gul and Atalar, 2019
*L. casei*	Freeze-dried	2.0, 3.0, and 4.0	0.5, 1, 2, and 3	Plate count enumeration	Gul and Atalar, 2019
*L. plantarum*	Freeze-dried	2	0.5, 1, 1.5, 2	Plate count enumeration	Tee et al., 2013
*L. reuteri*	Freeze-dried	2	30 min intervals	FC	Hernández et al., 2019
*L. reuteri*	Fresh cells	2	5, 15, 30, and 60 min	Plate count enumeration	Rosander et al., 2008

**TABLE 2:** A summary of the details of a collection of published bile tolerance assays.

Genus	Material	Concentration (%)	Type of bile	Duration (h)	Detection	References
*L. rhamnosus*	Freeze-dried	1	Porcine bile	1	Plate count enumeration	Stage et al., 2020
*L. casei*	Fresh cells	0.1	Cholate/deoxycholate	1	Plate count enumeration	Huang et al., 2018
Different LAB	Fresh cells	0.3	Ox bile salts	1, 2, 3, 4, 5, and 6	OD_600_	Ilango et al., 2016
Different LAB	Spray-dried	3.6	Ox bile salts	2 and 4	Plate count enumeration	Ilango et al., 2016
Different LAB	Freeze-dried	3.6	Ox bile salts	2 and 4	Plate count enumeration	Ilango et al., 2016
*B. animalis*	Fresh cells	0.3, 0.5, and 1	Ox bile salts	4	Plate count enumeration	Charnchai et al., 2016
*L. plantarum*	Spray-dried	2	–	1, 2, 3, and 4	Plate count enumeration	Dolly et al., 2011
*L. plantarum*	Freeze-dried	2	–	1, 2, 3, and 4	Plate count enumeration	Dolly et al., 2011
Different LAB	Fresh cells	0.3	Oxgall	0.5, 1, 1.5, 2, 3, 4, 5, 6, 7, and 24	OD_620_	Jacobsen et al., 1999
Different LAB	Fresh cells	0.3	Oxgall	4	Plate count enumeration	Jacobsen et al., 1999
Different LAB	Fresh cells	0.3, 0.6, 0.8, 1, 2, and 5	Oxbile	1, 2, 3, 4, 5, 6, 7, 8, 9, 10, 11	OD_600_	Brink et al., 2006
*L. casei*	Spray-dried	1, 2, and 3	Oxgall	0.5, 1, 2, and 3	Plate count enumeration	Gul and Atalar, 2019
*L. casei*	Freeze-dried	1, 2, and 3	Oxgall	0.5, 1, 2, and 3	Plate count enumeration	Gul and Atalar, 2019
*L. plantarum*	Freeze-dried	3	Taurocholic acid	4 and 8	Plate count enumeration	Tee et al., 2013
*L. reuteri*	Fresh cells	0.3	Oxgall	16	Continuous OD measurements	Taranto et al., 2003
*L. reuteri*	Freeze-dried	0.5	Porcine bile	30 min intervals	FC	Hernández et al., 2019
*L. reuteri*	Freeze-dried	1	Bovine bile	30 min intervals	FC	Hernández et al., 2019
*L. reuteri*	Fresh cells	6	Bovine bile	72	Plate count enumeration	Rosander et al., 2008

**TABLE 3:** A summary of the details of a collection of published pancreatin tolerance assays.

Genus	Material	Concentration (%)	Duration (h)	Detection	References
Different LAB	Spray-dried	0.1 (simulated gastric fluid)	2 and 4	Plate count enumeration	Ilango et al., 2016
Different LAB	Freeze-dried	0.1 (simulated gastric fluid)	2 and 4	Plate count enumeration	Ilango et al., 2016
*B. animalis*	Fresh cells	0.1	4	Plate count enumeration	Charnchai et al., 2016

**TABLE 4:** A summary of the details of a published lysozyme tolerance assay.

Genus	Material	Concentration (%)	Duration (h)	Detection	References
*L. plantarum*	Fresh cells	0.01	0.5 and 1.5	Plate count enumeration	Turchi et al., 2013

Except from the studies presented in Tables 1–4, a number of publications aim to assess the tolerance of passing through a simulated version of the complete gastrointestinal system to create a further realistic situation of the environment. Charnchai et al. (2016) tested freeze-dried *Bifidobacterium animalis* for its tolerance against simulated *in vivo* saliva conditions, the esophagus-stomach environment, and passage through the duodenum and ileum. Fredua-Agyeman and Gaisford exposed eight commercially available probiotic products to one kind of porcine gastric fluid and two kinds of simulated gastric fluids.

## Discussion

The growing interest in probiotics requires reliable methods for assuring the activity of the numerous products on the market (Kolaček et al., 2017; Hansen et al., 2018). Currently, the only information asked for by authorities are viability rates within a certain shelf-life in CFU, obtained from plate count enumeration (Hill et al., 2014; Fenster et al., 2019; Fiore et al., 2020; Hansen et al., 2020). Measuring CFU by plate count enumeration is one manner of assessing viability, but the concept is more complex than the ability to replicate. In this review, both viability and the ability to survive the passage through the human body and therefore be able to give a beneficial health effect are altogether defined as probiotic activity. The different methods, that are reviewed in this paper, each offers a piece of information contributing to a better understanding of probiotic activity.

One fundamental problem with methods based solely on culturability is their inability to detect all subpopulations that are included in the viable cell population (Iaconelli et al., 2015; Jackson et al., 2019). As mentioned before, several different divisions of a cell population based on viability markers have been published. Davis defined in 2014 four different non-culturable populations as the following: (1) non-replicating (active physiology and membrane integrity), (2) starving (dramatic decrease in metabolism), (3) dormant (low metabolic activity and inability to divide without additional recovery attempts, VBNC), and (4) irreparably damaged (progressively declining metabolism that finally terminates in death). Different methods have to be used for detection of these four populations. Therefore, to limit the definition of viability to culturability alone can be highly misleading and result in large underestimations of the number of active cells (Davey and Kell, 1996; Vinderola et al., 2019; Fiore et al., 2020).

Since label specifications on probiotic products describe viability numbers based on plate count enumeration, the question arises if the present non-culturable population not accounted for contributes to the overall health effect after consumption. Fiore et al. (2020) discussed the possible effects of both the presence of a VBNC population and the presence of dead cells in probiotic products, defining these two populations as “hidden microbial fractions.” Since microbial cells that are not able to grow on agar plates still can possess active metabolisms, there is a possibility that they can promote health benefits (de Almada et al., 2016). There is also a chance that dormant cells may reacquire the ability to reproduce once arriving in a favorable environment such as the gut (Senoh et al., 2012). In addition, non-viable cells, or parabiotics, have been indicated to promote health effects in a number of studies (Taverniti and Guglielmetti, 2011; Fujiki et al., 2012; Lahtinen, 2012; de Almada et al., 2016; Piqué et al., 2019). The ratio between the numbers of culturable cells and non-culturable cells will also change over time when stressed cells transfer to different non-culturable states, possibly moving the measured CFU further and further away from the actual viable cell count. As a result, there is a risk that the presence of this “hidden population” may affect the results of both stability studies and clinical trials on probiotics and consequently dose specifications. Today, a large collection of methods that have the abilities to reveal different viability characteristics are available. Culture-dependent methods reveal the ability to replicate but are reliant on optimized medium for the specific strain to be analyzed, thus often making strain separation impossible. In addition, culturability can be affected to a large extent by the suitability of the medium and previous stress exposure (García-Hernández et al., 2012; Olszewska et al., 2019; Vinderola et al., 2019). Among culture-independent methods, FC and other methods based on labeling with fluorescent probes, can reveal multiple important cell characteristics at the same time, such as membrane integrity, enzymatic activity, and metabolic activity (ISO, 2015). However, flow cytometric methods are as well often limited when it comes to strain separation. Other culture-independent methods based on molecular detection have the strength to be able to identify and separate probiotics on a strain level but are limited in the detection of viability markers. The use of PMA/EMA chemistry enables molecular technologies to combine strain-specific detection with assessment of membrane integrity (Rudi et al., 2005; García-Cayuela et al., 2009; Kramer et al., 2009; Gobert et al., 2018; Kiefer et al., 2020).

Since the herein described methods assess different viability markers, the number of detected viable cells can vary to a large extent between methods (Iaconelli et al., 2015; Vinderola et al., 2019). This aspect needs to be taken into consideration when comparing results from different analyses in stability studies and quality assurance. For example, a more rapid decrease in the number of active cells during storage has been seen with culturability analysis by plate count enumeration compared to membrane integrity analysis by FC (Foglia et al., 2020). In many cases, it is fruitless to strive for conformity between different methods, but instead see them as complementary to each other. Fiore et al. (2020) suggested that a total cell count by FC measurement should be added on probiotic product specifications to account for the populations not detected by plate count enumeration.

Although a probiotic culture has been determined to be viable after manufacturing, storage, and transportation, survival during its passage through the human body is desirable to enable positive health effects. The ability to tolerate exposure to the different stressors of the GIT have been assessed using many different assays designs. However, there is no established standard for assessing GIT stress tolerance and the link to *in vivo* survival is lacking.

It would be advantageous for the probiotic industry to rethink and extend the viability concept to an overall definition of activity that includes additional characteristics to culturability. Preferably, study designs can consist of a combination of several methods revealing a broader picture of probiotic activity—both characteristics indicating viability and tolerance against the stressors of the GIT.

It is crucial to carefully consider which kind of information that is desired to retrieve from a study design. Which activity markers are relevant for this particular sample and what are the limitations of the different methods available for their measurement? Are the results to be used in research or in quality assurance? In the case of stress tolerance assays, is the purpose to mimic the *in vivo* GIT environment with as high similarity as possible or is it to find a specific condition that will separate suitable from non-suitable probiotic cultures before product formulation?

To be able to compare results, it would be advantageous to define further standardized manners of assessing activity. However, a certain dynamic for strain dependence and type of sample has to be accounted for Vinderola et al. (2019). Assay results are often very strain dependent and affected by both sample type and formulation (Derrien and van Hylckama Vlieg, 2015; Iaconelli et al., 2015).

Apart from being able to conclude if a strain has the ability to maintain active throughout the manufacturing process, storage, and passage through the human body, it is of large importance to assess its ability to achieve beneficial health effects in the host. There are a number of available assays for assessing probiotic characteristics such as antimicrobial effects, the ability to colonize, and immunomodulatory characteristics (Papadimitriou et al., 2015). However, further assay development and standardization have to be performed to be able to mimic the complex intestinal environment *in vitro* and to be able to obtain reliable results.

As the probiotic field continues to evolve and flourish, the need for reliable methods for assessing probiotic activity has never been greater. With emerging technology, the possibilities of filling this need are extensive. The probiotic industry of tomorrow would greatly gain from an extended viability definition and standardized manners of identifying the complete activity state of a probiotic culture or product.

## Author Contributions

The author confirms being the sole contributor of this work and has approved it for publication.

## Conflict of Interest

UW was employed by company BioGaia AB.

## Publisher’s Note

All claims expressed in this article are solely those of the authors and do not necessarily represent those of their affiliated organizations, or those of the publisher, the editors and the reviewers. Any product that may be evaluated in this article, or claim that may be made by its manufacturer, is not guaranteed or endorsed by the publisher.
